# Wearable Biofeedback Improves Human-Robot Compliance during Ankle-Foot Exoskeleton-Assisted Gait Training: A Pre-Post Controlled Study in Healthy Participants †

**DOI:** 10.3390/s20205876

**Published:** 2020-10-17

**Authors:** Cristiana Pinheiro, Joana Figueiredo, Nuno Magalhães, Cristina P. Santos

**Affiliations:** Center for MicroElectroMechanical Systems (CMEMS), Department of Industrial Electronics, School of Engineering, University of Minho, 4800-058 Guimarães, Portugal; id6003@alunos.uminho.pt (J.F.); a68537@alunos.uminho.pt (N.M.); cristina@dei.uminho.pt (C.P.S.)

**Keywords:** ankle-foot exoskeleton, biofeedback control strategies, gait training, human-robot interaction, user-centered design, wearable biofeedback

## Abstract

The adjunctive use of biofeedback systems with exoskeletons may accelerate post-stroke gait rehabilitation. Wearable patient-oriented human-robot interaction-based biofeedback is proposed to improve patient-exoskeleton compliance regarding the interaction torque’s direction (joint motion strategy) and magnitude (user participation strategy) through auditory and vibrotactile cues during assisted gait training, respectively. Parallel physiotherapist-oriented strategies are also proposed such that physiotherapists can follow in real-time a patient’s motor performance towards effective involvement during training. A preliminary pre-post controlled study was conducted with eight healthy participants to conclude about the biofeedback’s efficacy during gait training driven by an ankle-foot exoskeleton and guided by a technical person. For the study group, performance related to the interaction torque’s direction increased during (*p*-value = 0.07) and after (*p*-value = 0.07) joint motion training. Further, the performance regarding the interaction torque’s magnitude significantly increased during (*p*-value = 0.03) and after (*p*-value = 68.59 × 10^−3^) user participation training. The experimental group and a technical person reported promising usability of the biofeedback and highlighted the importance of the timely cues from physiotherapist-oriented strategies. Less significant improvements in patient–exoskeleton compliance were observed in the control group. The overall findings suggest that the proposed biofeedback was able to improve the participant-exoskeleton compliance by enhancing human-robot interaction; thus, it may be a powerful tool to accelerate post-stroke ankle-foot deformity recovery.

## 1. Introduction

Gait disabilities are mainly caused by stroke, affecting the quality of life of 13 million people per year worldwide [1,2]. The ankle-foot deformity is the most detrimental abnormality for gait ability post-stroke, with an incidence of 10–20% [3]. As a consequence, post-stroke patients adopt compensatory, energetically inefficient gait patterns, leading to professional and social exclusion and increased risk of falling [1,3].

Stroke survivors may recover their motor ability through gait rehabilitation. Gait rehabilitation driven by exoskeletons allows intensive, user-adaptive, and repetitive gait training; thus, increasing effectiveness and accelerating long-term recovery [4]. This gait rehabilitation follows a bottom-up rehabilitative approach, aiming to influence the central nervous system (top)—neuroplasticity—through actuation on the lower limbs (bottom) [5].

Biofeedback systems (BSs) are robotic devices that measure gait-related unconscious parameters through sensors and feedback this information to users in real-time through visual, auditory, and/or haptic cues, using appropriate actuators [6]. Through the BSs, patients may be aware of their abnormal motor behavior and they are intensively and repetitively encouraged to self-control it towards recovery [6]. Complementary to exoskeletons, BSs support a top-down rehabilitative approach, exerting direct action on the central nervous system to recover peripheral functions [5]. The adjunctive use of BSs during gait rehabilitation driven by exoskeletons can accelerate post-stroke long-term motor recovery once the BSs potentiate neuroplasticity by encouraging patients’ active motor relearning and preventing patient’s motor dependence on the exoskeleton [5].

Physiotherapist-oriented control strategies can be coupled to BSs so physiotherapists can assess patient’s performance easily and objectively during the therapy. This is particularly important during gait rehabilitation driven by exoskeletons once physiotherapists cannot have the traditional physical contact with the patient’s limbs [7]. Physiotherapists’ involvement during therapy allows additional support to patients, assuring safe and effective use of the robotic devices, and fostering patient’s motivation [8].

Some studies are reporting the promising use of biofeedback during post-stroke gait rehabilitation driven by exoskeletons. Most of them include gait training driven by hip and/or knee exoskeletons (EMG feedback in [9] and human-robot interaction torque feedback in [10]). EMG feedback is provided through a non-wearable display of colored stripes (one stripe per muscle) portioned into 16 stages within the gait cycle [9]. The stripes’ color represents over-activation, under-activation, or optimal muscle contraction according to a reference dataset [9]. Muscle contraction is measured through electromyographic sensors that require a time-consuming preparation. The human-robot interaction torque feedback is given by a non-wearable display of a smiley character with a mouth’s length and shape modulated according to the averaged human-robot interaction torque and a pre-defined threshold, respectively [10]. Human-robot interaction torque is measured by force sensors embedded in the exoskeleton’s structure and it does not require any preparation. Moreover, human-robot interaction torque feedback was showed to be more effective to improve patient-exoskeleton compliance than EMG feedback [9].

Additionally, the study [10] proposed control strategies to foster the physiotherapist’s involvement during gait rehabilitation driven by exoskeletons through a physiotherapist-oriented, non-wearable display of the patient-exoskeleton interaction torque. The display includes technical graphs for hip and knee joints, and swing and stance phases [10].

Only one study reported the use of biofeedback with an ankle-foot exoskeleton, using a game based on measured ankle angle to encourage patients to perform simple dorsiflexion and/or plantar flexion movements while sitting (which is not functional gait training) [11]. A gyrocopter character is displayed on a non-wearable screen and moves upwards/downwards according to the user’s dorsiflexion/plantar flexion movements that should follow the trajectory delineated through gas bottle characters [11].

Based on actual literature advances, it is still needed to develop (i) biofeedback control strategies for functional gait training driven by ankle–foot exoskeletons to accelerate motor recovery of post-stroke patients with an ankle-foot deformity; (ii) control strategies to foster physiotherapist involvement for additional patient support; and (iii) fully wearable biofeedback solutions to enable ambulatory use and daily practice.

This work proposes two novel biofeedback control strategies—joint motion and user participation strategies—to complement gait rehabilitation driven by ankle-foot exoskeletons by improving patient–exoskeleton compliance; this so that patients can relearn to perform a healthy gait pattern, regaining their quality of life. Wearable auditory and vibrotactile cues are provided every time that the user interacts with the exoskeleton in the opposite direction to (joint motion strategy) and following (user participation strategy) the exoskeleton’s direction of movement, respectively. The joint motion strategy aims to encourage users to not interact with the exoskeleton in the opposite direction to the exoskeleton’s direction of movement. The user participation strategy encourages users to reduce the magnitude of the interaction torque. Parallel strategies are additionally proposed so a physiotherapist can follow in real-time the patient’s gait training through wearable visual cues. The biofeedback is supplied by a wearable system, able to be used with different exoskeletons due to its open-architecture and to provide multimodal cues. It includes visual cues from a Light-Emitting Diode oriented to the physiotherapists, and auditory and vibrotactile cues from an earphone and elastic vibrotactile shank bands, respectively, both oriented to the patients. Detailed information about the design of the used BS can be found in [12].

A preliminary pre-post study design was performed to prove the concept with healthy subjects, using a fully wearable BS and a unilateral (right side) ankle-foot exoskeleton. This experimental evaluation allowed to pursue the following research question: “Are BSs able to improve human-robot compliance during gait training driven by ankle-foot exoskeletons?”. This study contributes by innovatively providing research evidence concerning the promising use of BSs during gait training driven by ankle-foot exoskeletons through human-robot interaction-based control strategies involving patient and physiotherapist-oriented wearable cues.

## 2. Methods

### 2.1. Trajectory Tracking Control of an Ankle–Foot Exoskeleton

The proposed biofeedback is applied during gait training driven by a trajectory tracking controlled ankle-foot exoskeleton [13]. This robotic device has a flexion/extension range of motion of 20° and it is composed of an electrical actuator (flat brushless DC motor EC60-100W, Maxon) coupled to a gearbox (CSD20-160-2A strain wave gear, Harmonic Drive) that provide a continuous net torque of 35 Nm and peak torque of 180 Nm. It also includes the following sensors: (i) a precision potentiometer (resolution of 0.5°) as the angle position sensor; (ii) four strain gauges connected to a full Wheatstone bridge to measure the human-exoskeleton interaction torque with a resolution of 1 Nm; and (iii) two force resistive sensors at the heel and toe to measure the ground reaction force. More details concerning the overall exoskeleton design are presented in [14].

The exoskeleton follows a bioinspired hierarchical control architecture involving three levels (low-level, mid-level, and high-level). The high-level control runs at 100 Hz on a Raspberry Pi 3 (Raspberry Pi Foundation, UK) and includes a reference angle generator. It adjusts the healthy ankle sagittal plane gait angle reported by Winter in [15] according to gait speed. This adjustment follows Equation (1), where *s* is the gait speed from 0.5 km/h to 1.6 km/h and *t* is the time in ms between each point of the reference angle. This trajectory is set as the ankle-foot exoskeleton reference joint angle by the mid-level controller. The low-level control is based on a close-loop proportional-integral-derivative (PID) controller described in [16]. The mid-level and low-level control ran at 1000 Hz on an STM32F4-Discovery board (STMicroelectronics, Switzerland).

**Figure 1:**
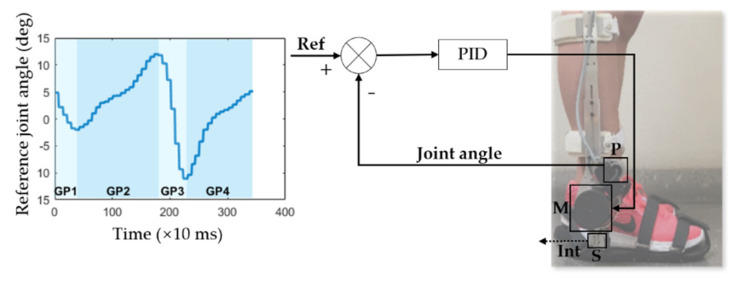
Diagram of the ankle–foot exoskeleton’s trajectory tracking control, where *Ref* is the reference joint angle (segmented in four gait phases GP1-4), *Int* is human-robot interaction torque, *M* is the actuator, *P* is the potentiometer, and *S* is the strain gauge.

(1) t = −34.62×s+107.31,

As can be seen in Figure 1, the output of the PID controller acts on the exoskeleton’s actuator *M*, generating torque. Since the exoskeleton is coupled to the user’s limb, the actuator’s torque is applied on the user’s ankle to follow the reference joint angle (*Ref*). According to the controller, the actuator acts every time that the real joint angle is not equal to the reference joint angle. The difference between the real and reference joint angle can result from the effect of the human-robot interaction torque (*Int*) in the joint angle.

Human-robot interaction torque comprises moments generated by gravitational, inertial, viscoelastic, and muscular forces applied to the exoskeleton. A baseline interaction torque is measured when the user does not interact with the exoskeleton, due to the exoskeleton’s gravitational and inertial components, indicating that the user is compliant with the robot and actively follows the reference joint angle. On the other hand, interaction torque departs from the baseline (increases/decreases) when the user interacts with the exoskeleton, applying a force on it (towards plantar flexion/dorsiflexion) that will be compensated for by the exoskeleton’s actuator to follow the reference joint angle.

### 2.2. Wearable, Multimodal, and Open-Architecture Biofeedback System

**Figure 2:**
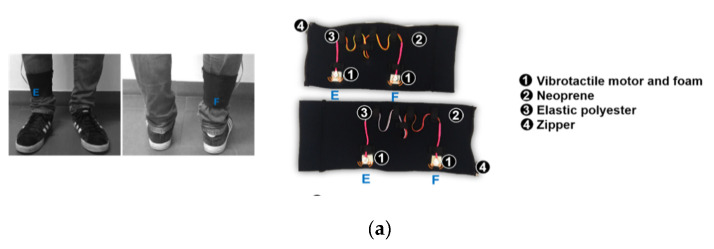

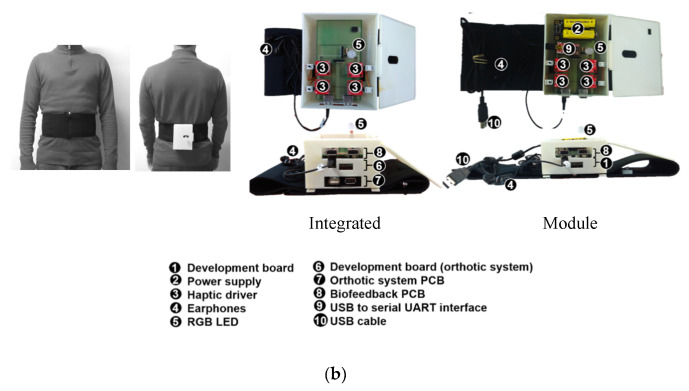
(**a**) Vibrotactile shank bands and (**b**) BS components and electronic interfaces when BS is used integrated into or as a module of the ankle-foot exoskeleton.

The proposed human-robot interaction-based biofeedback strategies were implemented into a wearable, multimodal, and open-architecture BS (Figure 2), as described in [12]. It can provide patient-oriented vibrotactile and auditory cues through Eccentric Rotating Mass motors placed on elastic waist and shank bands, and earphones, respectively. Moreover, the BS can provide physiotherapist-oriented visual cues through a Red-Green-Blue Light-Emitting Diode placed on the patient’s back so it can only be perceived by the physiotherapist. An STM32F4-Discovery board manages the activation of these cues in real-time according to the ankle-foot exoskeleton data (i.e., reference joint angle and human-robot interaction torque). The versatility of the BS allows its use as integrated (the BS uses the exoskeleton’s development board and power supply) or as a module (the BS has its development board and power supply and communicates with the exoskeleton through a serial UART interface) of the ankle-foot exoskeleton (Figure 2b). Its open-architecture allows its use with different exoskeletons.

### 2.3. Human-Robot Interaction-Based Biofeedback Strategies

According to a healthy gait pattern, the ankle joint performs two types of movements in the sagittal plane: dorsiflexion (ankle rotation against gravity) and plantar flexion (ankle rotation in favor of gravity), leading to the gait phases indicated in Figure 1 [17]. These movements occur through appropriate contraction of specific muscles [17]. Human-robot interaction comprises moments generated by muscular forces applied to the exoskeleton. Two biofeedback strategies are proposed based on filtered human-robot interaction torque (through a 2nd order Butterworth low-pass filter at 5 Hz): joint motion and user participation strategies.

#### 2.3.1. Joint Motion Strategy

Joint motion biofeedback strategy encourages users to self-control their compliance with the exoskeleton’s direction of movement (dorsiflexion and plantar flexion) by not interacting with the opposite direction to the exoskeleton’s direction of movement. Through an empiric analysis with the used ankle–foot exoskeleton, it was possible to conclude when the user is not compliant with the exoskeleton’s direction of movement by analyzing the relationship among the human-robot interaction torque and reference joint angle, as follows: when reference joint angle increases/decreases (exoskeleton’s dorsiflexion/plantar flexion) and human-robot interaction torque is bigger/smaller than the baseline (user interacts with the exoskeleton towards plantar flexion/dorsiflexion).

Therefore, during this strategy, an auditory cue is enabled when the user is not compliant with the exoskeleton’s direction of movement. The auditory cue is a sinusoidal tone (200 Hz) provided through a single earphone included in the BS [12]. The volume was adjusted so that users can hear the physiotherapist’s verbal instructions for additional support. The strategy is applied during one of the gait phases (GPs) presented in Figure 1. The training gait phase corresponds to the gait phase, presented in Figure 1, in which the user achieved the lowest mean performance regarding the direction of the human-robot interaction (Performance Dir) during three pre-training trials. The gait phases were calculated in real-time according to the generated reference joint angle (which comprises a 48 values-array), as follows: GP1 was identified from the 1st to the 5th value of the reference joint angle; GP2 from the 6th to the 24th; GP3 from the 25th to the 31st; and GP4 from the 32nd to the 48th value. The 1st, 5th, 24th, and 31st values correspond to the heel-strike, flat-foot, heel-off, and toe-off gait events according to Winter in [15]. The study by [12] shows the operability of this algorithm. Each pre-training trial involves a 1-min walk on a treadmill with the ankle-foot exoskeleton running the trajectory tracking control at the training gait speed. The Performance Dir is calculated for each gait phase through Equations (2) and (3), where n is the gait phase’s total number of samples, Ref (i) is reference joint angle at sample i (100 Hz sample frequency), Int (i) is the human-exoskeleton interaction torque at sample i (100 Hz sample frequency), and Bas is the baseline interaction torque. This value was empirically defined as the measured human-robot interaction torque when the ankle-foot exoskeleton runs the trajectory tracking control suspended in the air without a user (varies from −2 Nm to 2 Nm). Thus, it comprises moments generated by gravitational and inertial forces exerted by the exoskeleton itself. A *Performance Dir* equal to 100% means that the user does not interact with the exoskeleton or interacts with a direction that is following the exoskeleton’s direction of movement throughout the gait phase.

(2) $$\;Performance\;Dir\;(\%)\;=\;\frac{\sum_{i\;=\;2}^{n}x}{n}\;\times \;100,$$

(3) $$\;x\left(i\right)\;=\;\{\begin{array}{c} 1\;\mathrm{if}\;\left[Ref\left(i\right)-Ref\left(i-1\right)\;>\;0\;\mathrm{and}\;Int\left(i\right)\;<\;Bas\right]\;\mathrm{or}\;[Ref\left(i\right)-Ref\left(i-1\right)\;<\;0\;\mathrm{and}\;Int\left(i\right)\;>\;Bas]\\ 0\;\mathrm{otherwise} \end{array},$$

This strategy was designed to maximize the user’s satisfaction regarding the system’s usability (user-centered design) and to involve a low cognitive effort once (i) the user only needs to distinguish the on and off state of the auditory cue; (ii) the auditory cue is a simple tone; and (iii) the strategy is applied during only one gait phase according to the user’s imminent needs, allowing a low cognitive effort and personalized gait training, respectively.

#### 2.3.2. User Participation Strategy

The user participation biofeedback strategy encourages the users to self-control their interaction, in terms of magnitude, with the exoskeleton, to be compliant with the exoskeleton’s movement. The user is not compliant with the exoskeleton when the magnitude of the interaction is departing from the baseline, indicating that the user is not contracting her/his muscles as specified by the healthy gait pattern. A user-adaptive threshold was created to relieve the gait training difficulty level according to the patient’s disability such that the human-robot interaction torque can vary between the baseline and such a threshold.

The threshold is defined according to the mean user’s performance during three pre-training trials. The pre-training trial involves a 1-min walk on a treadmill with the ankle-foot exoskeleton running the trajectory tracking control at the training gait speed. The user-adaptive threshold module (|Thr|) is calculated through Equation (4) using the data relative to the gait phase addressed during training (training gait phase—described below), where n is the training gait phase’s total number of samples and Int is the human-exoskeleton interaction torque.

(4) $$|Thr|\;=\;\frac{\max(|Int(1:n)|)}{2},$$

During this strategy, a vibrotactile cue is enabled when the user interacts with the exoskeleton and the direction of interaction is following the exoskeleton’s direction of movement. The vibrotactile cue is provided at 200 Hz 1.8 G through a vibrotactile shank band included in the BS [12]. This frequency was successfully perceived by post-stroke patients in [18]. The vibrotactile motor placed on the front (near tibialis anterior muscle) and back (near soleus muscle) of the shank is enabled when the interaction occurs during the exoskeleton’s dorsiflexion and plantar flexion, respectively. Therefore, the cue is provided near to the main muscle that needs to be controlled to achieve human-robot compliance. As the joint motion strategy, this strategy is applied during one of the gait phases (GPs) presented in Figure 1. The training gait phase corresponds to the gait phase, presented in Figure 1, in which the user achieved the lowest mean performance regarding the magnitude of the human-robot interaction (Performance Mag) during the three pre-training trials. The Performance Mag is calculated for each gait phase through Equations (5) and (6), where n is the gait phase’s total number of samples, Int is the human-exoskeleton interaction torque, and Bas is the baseline interaction torque. A *Performance Mag* equal to 100% means that the user did not interact with the exoskeleton throughout the gait phase.

(5) $$Performance\;Mag\;(\%)\;=\;\frac{\sum_{i\;=\;1}^{n\;}y}{n}\;\times \;100,$$

(6) $$\;y\left(i\right)\;=\;\{\begin{array}{c} 1\;\mathrm{if}\;Int\left(i\right)\in Bas\\ 0\;\mathrm{otherwise} \end{array},$$

This strategy was developed following a user-centered design once (i) the user only needs to distinguish the on and off state of the vibrotactile cue; (ii) it is applied during only one gait phase according to the user’s imminent needs; (iii) the vibrotactile cue is intuitively provided near to the main muscle that needs to be controlled to achieve human-robot compliance; and (iv) a user-adaptive threshold is calculated according to user’s disability, allowing a low cognitive effort and personalized training.

### 2.4. Physiotherapist-Oriented Control Strategies

The physiotherapist-oriented control strategies run in parallel with biofeedback strategies to allow the physiotherapists to assess users’ performance during gait training. Visual cues, red and green lights, are provided during the training gait phase through an RGB LED included in the BS [12], intuitively indicating low and high user performance, respectively. During the joint motion biofeedback strategy, a red light automatically appears when the user interacts with the exoskeleton with a direction opposite to the exoskeleton’s direction of movement. On the other hand, a green light automatically appears when the user does not interact with the exoskeleton or when the user interacts with the exoskeleton in a direction that is following the exoskeleton’s direction of movement. During the user participation biofeedback strategy, red and green lights automatically appear when the user does (but following the exoskeleton’s direction of movement) and does not interact with the exoskeleton, respectively. In this manner, physiotherapists can easily and objectively follow the user’s motor behavior during gait training driven by the exoskeleton and, consequently, provide effective additional support, aiming for long-term effective recovery.

### 2.5. Experimental Validation

#### 2.5.1. Participants

Eight healthy subjects (without any history of neuromuscular and musculoskeletal impairments) from the Center of MicroElectroMechanical Systems of the University of Minho were systematically recruited to participate in a pre-post controlled study to evaluate the efficacy of the proposed biofeedback strategies for improving human-robot compliance during gait training driven by an ankle-foot exoskeleton. All subjects gave their informed consent for inclusion before their participation in the study. The study was conducted following the Declaration of Helsinki, and the protocol was approved by the Ethics Committee CEICVS 006/2020. The participants were assigned to experimental (age: 25 ± 1 years; body mass: 65 ± 12 kg; and body height: 1.69 ± 0.13 m) and control (age: 24 ± 0 years; body mass: 71 ± 6 kg; and body height: 1.74 ± 0.05 m) groups. All participants were familiarized with the trajectory tracking control of the ankle-foot exoskeleton. Table 1 presents the gender, age, and anthropometric characteristics (body mass and height) of all participants.

#### 2.5.2. Experimental Protocol

**Figure 3:**
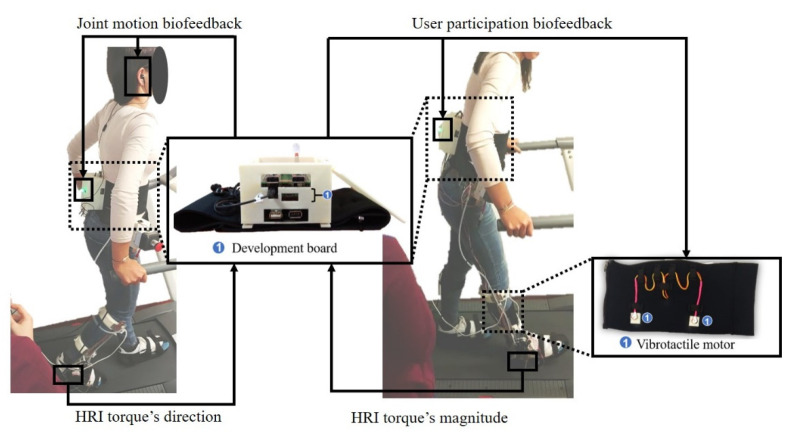
Pictures of participant 1 and technical person during joint motion and user participation biofeedback training with indication of the used cue (visual and auditory or vibrotactile) and its meaning (HRI torque’s direction or magnitude).

The protocol performed by the experimental group includes three procedures for each biofeedback strategy englobing a total of eight walking trials (17 gait cycles per trial): (1) pre-training (three trials) (PRTR); (2) training (four trials including a familiarization trial) (TR); and (3) post-training (one trial) (PSTR). PRTR involved the execution of three trials walking on a treadmill at 1 km/h for 1 min (17 gait cycles/trial) with the ankle-foot exoskeleton running trajectory tracking control. The participants were instructed to actively follow the exoskeleton’s movement. This procedure allows the configuration of the biofeedback strategies (namely, the training gait phase and threshold) according to the user’s imminent needs. Then, the participants performed one familiarization trial with the BS as a complementary tool to the ankle-foot exoskeleton gait training. This trial lasted until the participants felt prepared for the biofeedback training. The maximum duration registered was 2 min and 5 min for joint motion and user participation biofeedback strategies, respectively. Subsequently, we asked the participants to conduct the TR procedure (Figure 3) that was similar to PSTR with the addition of the BS. The participants trained, firstly, the joint motion strategy, and then the user participation strategy. Thus, for effective learning, the participants may learn, firstly, to follow the exoskeleton’s direction of movement, and then the magnitude of the interaction. PSTR consists of a retention trial performed 2 min after the TR procedure. The participants were challenged to replicate the trained gait pattern without the BS’s cues for 1 min. A gait speed of 1 km/h was selected to perform the experiments since it is the intermediate gait speed at the range of the ankle–foot exoskeleton’s assistance gait speeds and is tailored for target end-users (post-stroke patients), as shown in [14]. Moreover, a 1 km/h walking speed was already successfully used with the same exoskeleton in a healthy population [19]. A technical person with enough experience in gait training driven by an ankle–foot exoskeleton and BS supported the experimental group mainly during the TR procedure (the second person in Figure 3) by providing instructions according to the visual cues from the physiotherapist-oriented control strategies. During joint motion training (user participation strategy), the participants were instructed to start or end (perform) dorsiflexion or plantar flexion early (faster) or later (slower).

The control group performed the same procedures (during the same time, at the same walking speed, with the ankle–foot exoskeleton running trajectory tracking the control) without the BS to control the effects of the exoskeleton use regarding the outcomes of interest. The participants were instructed to actively follow the exoskeleton’s movements without any cue from the BS, nor verbal instructions from the technical person.

Both the experimental and the control groups were instructed before the pre-training procedure by the technical person to walk with the exoskeleton with minimum interaction, following actively the exoskeleton’s movement. The experimental group received additional instructions only concerning the meaning of the biofeedback cues, which is indispensable for the success of the biofeedback training. Therefore, the technical person instructed the participants to stop their interaction with the exoskeleton against its direction of movement when the auditory cue is perceived. Additionally, the participants were instructed to stop their interaction with the exoskeleton when the vibrotactile cue is perceived.

#### 2.5.3. Data Collection and Processing

The reference joint angle, real joint angle, and human-robot interaction torque were acquired at 100 Hz during all procedures (excluding familiarization trial) through the embedded sensors in the exoskeleton. The mean and standard deviation of the following metrics were calculated per experimental procedure: (M1) the maximum and minimum human-robot interaction torque; (M2) the Root-Mean-Square (RMS) of the human-robot interaction torque for the training gait phase and gait cycle (gait phases and gait cycle were segmented through reference joint angle data, as shown in Figure 1); (M3) the delay between the reference joint angle and real joint angle; (M4) the Root-Mean-Square-Error (RMSE) between the reference joint angle and real joint angle for the training gait phase and gait cycle; and (M5) the user’s performance for the training gait phase and gait cycle regarding the direction (Equations (2) and (3)) and the magnitude (Equations (5) and (6)) of the human-robot interaction, and magnitude of the human-robot interaction with a threshold (Performance Mag Thr) (Equations (7) and (8)), where n is the gait phase’s total number of samples, Ref is the reference joint angle, Int is the human-exoskeleton interaction torque, Bas is the baseline interaction torque, and |Thr| is the training threshold module (Equation (4)). A *Performance Mag Thr* equal to 100% means that the user reduced the interaction with the exoskeleton according a pre-defined user-adaptive threshold throughout the gait phase.

(7) $$\;Performance\;Mag\;Thr\;(\%)\;=\;\frac{\sum_{i\;=\;2}^{n}z}{n}\times 100,$$

(8) $$\;z\left(i\right)\;=\;\{\begin{array}{c} 1\;\mathrm{if}\;\left[Ref\left(i\right)-Ref\left(i-1\right)>0\;\mathrm{and}\;Bas\geq Int\left(i\right)\geq -Thr\right]\;\mathrm{or}\;[Ref\left(i\right)-Ref\left(i-1\right)<0\;\mathrm{and}\;Bas\leq Int\left(i\right)\leq Thr]\\ 0\;\mathrm{otherwise} \end{array},$$

The training gait phase and threshold were also determined for each participant of both the experimental and control group immediately after the pre-training procedure. For the control group, the data from the first experimental procedure were considered to obtain equivalent metrics between the participants of the experimental and control groups. The first and last three gait cycles of each trial were discarded from processing. There were no withdrawals nor dropouts.

Shapiro–Wilk tests were performed to evaluate the normal distribution of the data. Six paired two-sided *t*-tests and Wilcoxon Signed Rank tests were performed per parametric and non-parametric independent metrics, respectively, considering a significance level of 0.05 and the following null hypotheses. There are no statistically significant differences between the (i) PRTR and TR of the joint motion biofeedback strategy by the experimental group; (ii) PRTR and PSTR of the joint motion biofeedback strategy by the experimental group; (iii) PRTR and TR of the user participation biofeedback strategy by the experimental group; (iv) PRTR and PSTR of the user participation biofeedback strategy by the experimental group; (v) PRTR and TR by the control group; and (vi) PRTR and PSTR by the control group.

At the end of the protocol, the experimental group was asked to comment on the biofeedback training and to answer a questionnaire regarding the BS’s usability considering both biofeedback strategies. The questionnaire was based on the System Usability Scale (Digital Equipment Corporation, United Kingdom) that provides a global view of a system’s usability subjective assessment [20]. The participants chose “strongly agree”, “agree”, “no opinion”, “disagree”, or “strongly disagree” regarding the following sentences: (Q1) “I think that I can walk more compliantly with the exoskeleton after using the BS”; (Q2) “I need more time to learn how the BS works before I can get going with it”; (Q3) “I need to learn other things before I can get going with the BS”; (Q4) “I imagine that most people will learn to use the BS very quickly”; and (Q5) “After understanding how the BS works, I think that I need the support of a physiotherapist along with gait training”. The modal answer (answer reported by most participants) was calculated for each usability sentence. Additionally, a technical person who followed the participants during training commented on the physiotherapist-oriented strategies.

## 3. Results

Table 2 shows the training gait phases for both biofeedback strategies (modal training gait phase: 3 for joint motion and 1 for user participation strategies) and the training threshold module (4 ± 1 Nm) for user participation strategy.

**Figure 4:**
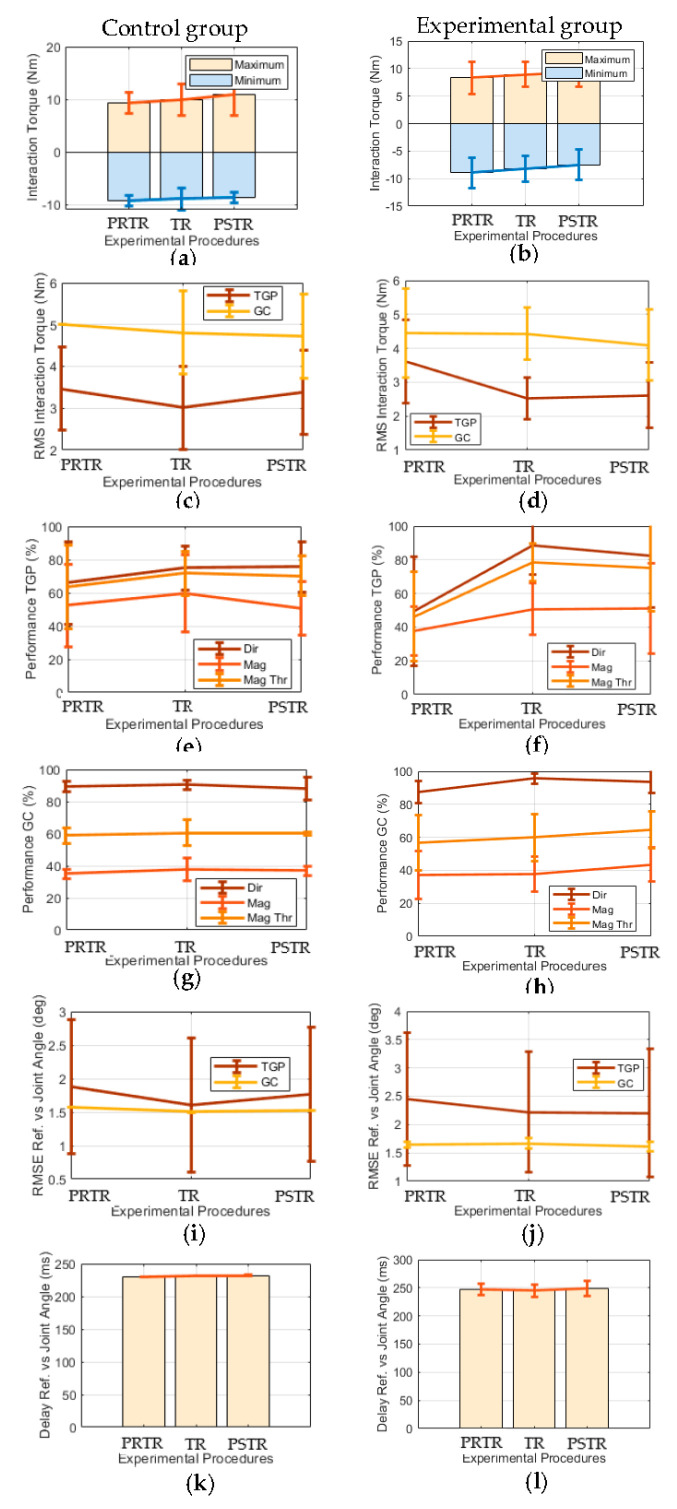
Mean and standard deviation per procedure (PRTR, TR, and PSTR) of (**a**,**b**) maximum and minimum human-robot interaction torque; (**c**,**d**) the RMS of the interaction torque for the training gait phase (TGP) and overall gait cycle (GC); *Performance Dir, Mag, Mag Thr* for (**e**,**f**) TGP and (**g**,**h**) GC; (**i**,**j**) the RMSE for TGP and GC; and (**k**,**l**) the delay between the reference joint angle (*Ref*) and real joint angle for the control/experimental group regarding the joint motion biofeedback strategy.

Figure 4 illustrates the mean and standard deviation of the acquired metrics (M1–M5) per experimental procedure for both the experimental and control groups (Figure S1 and Table S1 present complementary results individualized by each participant) regarding the joint motion biofeedback strategy. Table 3 complements these results by presenting the *p*-values of the statistical tests for each metric and group between PRTR and TR and PRTR and PSTR. As can be seen in Table 3, there were no statistically significant results for both groups regarding the joint motion biofeedback strategy. However, the most representative results (*p*-value < 0.10) of the experimental group indicate greater improvements than the control group in the following metrics: (i) *Performance Dir* increased for training gait phase between PRTR and TR (*p*-value = 0.07 vs. *p*-value = 0.27) and between PRTR and PSTR (*p*-value = 0.07 vs. *p*-value = 0.26), and for gait cycle between PRTR and TR (*p*-value = 0.07 vs. *p*-value = 0.31); (ii) *Performance Mag* increased for gait cycle between PRTR and PSTR (*p*-value = 5.9 × 10^−2^ vs. *p*-value = 0.34); and (iii) *Performance Mag Thr* increased for the training gait phase between PRTR and TR (*p*-value = 0.09 vs. *p*-value = 0.30) and between PRTR and PSTR (*p*-value = 0.07 vs. *p*-value = 0.39).

**Figure 5:**
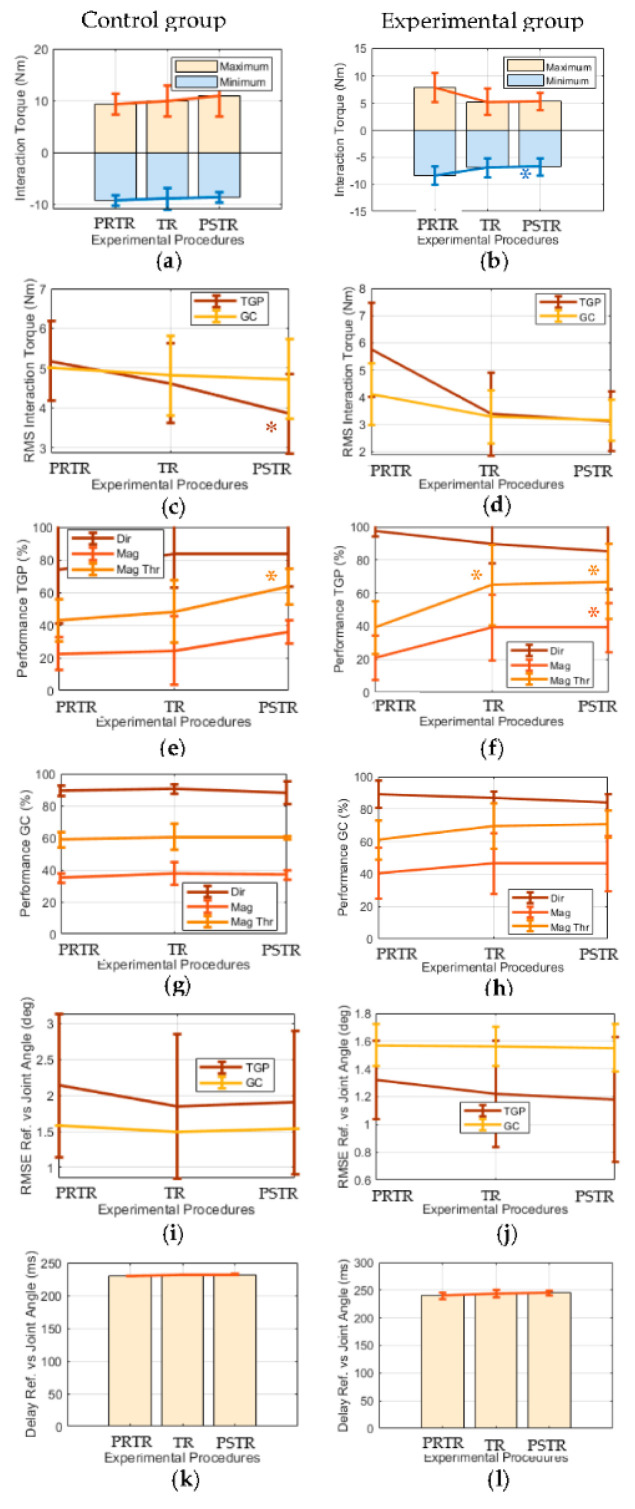
Mean and standard deviation per procedure (PRTR, TR, and PSTR) of (**a**,**b**) the maximum and minimum human-robot interaction torque; (**c**,**d**) the RMS of the interaction torque for the training gait phase (TGP) and overall gait cycle (GC); *Performance Dir, Mag, Mag Thr* for (**e**,**f**) TGP and (**g**,**h**) GC; (**i**,**j**) the RMSE for TGP and GC; and (**k**,**l**) the delay between the reference joint angle (*Ref*) and real joint angle for the control/experimental group regarding the user participation biofeedback strategy. The symbol “*” means a statistically significant result of the statistical test between PRTR and TR/PSTR.

Figure 5 shows the mean and standard deviation of the computed metrics (M1–M5) per procedure in user participation biofeedback strategy for the experimental and control groups (Figure S1 and Table S2 present complementary results individualized by each participant). Table 4 complements these results presenting the *p*-values of the statistical tests for each metric and group between PRTR and TR and PRTR and PSTR. Regarding the user participation biofeedback strategy, Table 4 demonstrates that the experimental group increased significantly and the control group non-significantly: (i) the minimum interaction torque between PRTR and PSTR (*p*-value = 0.04 vs. *p*-value = 0.14); (ii) the *Performance Mag Thr* for training gait phase between PRTR and TR (*p*-value = 0.03 vs. *p*-value = 0.53); and (iii) the *Performance Mag* for training gait phase between PRTR and PSTR (*p*-value = 4.56 × 10^−4^ vs. *p*-value = 0.08). Moreover, the experimental group increased more significantly than the control group the *Performance Mag Thr* for the training gait phase between PRTR and PSTR (*p*-value = 8.59 × 10^−3^ vs. *p*-value = 0.01).

Additionally, the most representative results (*p*-value < 0.10) of the experimental group also include greater improvements than the control group in the following metrics: (i) minimum interaction torque increased between PRTR and TR (*p*-value = 0.07 vs. *p*-value = 0.46); (ii) the RMS of the interaction torque decreased for the training gait phase between PRTR and TR (*p*-value = 0.07 vs. *p*-value = 0.32) and for the gait cycle between PRTR and PSTR (*p*-value = 0.08 vs. *p*-value = 0.19); (iii) *Performance Mag* increased for the training gait phase between PRTR and TR (*p*-value = 5.57 × 10^−2^ vs. *p*-value = 0.77); and (iv) *Performance Mag Thr* increased for the gait cycle between PRTR and TR (*p*-value = 5.50 × 10^−2^ vs. *p*-value = 0.65) and between PRTR and PSTR (*p*-value = 0.08 vs. *p*-value = 0.68). On the other hand, the *Performance Dir* decreased and increased for the training gait phase between PRTR and TR of experimental and control groups, respectively (*p*-value = 0.07, *p*-value = 0.20).

As can be perceived by analyzing Figure 2 and Figure 3, the control group presents similar results to the experimental group concerning the PT procedure for all metrics and both biofeedback strategies.

Table 5 presents the comments of the participants from the experimental group regarding the biofeedback training with joint motion and user participation strategies, and comments of the technical person who followed the participants during the experimental protocol related to physiotherapist-oriented control strategies. In general, the participants referred to that (i) the biofeedback (joint motion and user participation biofeedback strategies) increased their motivation to perform the gait training; (ii) they felt more compliant with the exoskeleton after the use of the biofeedback; (iii) they demonstrated a positive appreciation concerning the auditory and vibrotactile cues; and (iv) they felt confident to achieve a better performance with longer biofeedback training. The technical person commented on the promising use of physiotherapist-oriented control strategies once (i) it is very difficult to perceive the human-robot compliance through visual inspection; and (ii) the visual cues from the BS are intuitive, enabling timely instructions for guiding the user’s motor performance.

Table 6 displays the answers of the experimental group concerning the usability questionnaire (Q1–Q5) about both biofeedback strategies. Most of the participants strongly agreed/agreed with the sentence “I think that I can walk more compliantly with the exoskeleton after using the BS”, agreed that “I need more time to learn how the BS works before I can get going with it”, disagreed with the sentence “I need to learn other things before I can get going with the BS”, and agreed with the sentence “After understanding how the BS works, I think that I need the support of a physiotherapist along with gait training”. However, there is no consensus concerning the timing needed to learn how to use the BS, as the participants equally reported that they agreed and disagreed with the sentence “I imagine that most people will learn to use the BS very quickly”.

## 4. Discussion

This work proposes two novel human-robot interaction-based biofeedback strategies to complement gait rehabilitation driven by ankle–foot exoskeletons following the hypothesis that human-robot interaction-based biofeedback can improve patient compliance with the exoskeleton; thus, relearning to perform a healthy gait pattern. Therefore, the timing for motor recovery of post-stroke patients with ankle-foot deformities is accelerated, and they more quickly regain quality of life. This hypothesis is also followed by [10] with hip and knee exoskeletons, and it is reinforced by [9], concluding that human-robot interaction-based biofeedback is more effective than EMG-based biofeedback to improve patient’s compliance with the robot movement. Contrarily to [9,10,11], the proposed strategies followed a user-centered design, allowing to personalize the biofeedback (namely, the training gait phase and training threshold) according to the user’s imminent needs; thus, aiming for low cognitive effort and user satisfaction. Moreover, contrarily to [9,10,11], the proposed biofeedback strategies and physiotherapist-oriented control strategies are implemented in a fully wearable BS and ankle-foot exoskeleton, allowing ambulatory use and daily practice.

A pre-post controlled study was performed with healthy participants to conclude about the potential of the proposed biofeedback strategies to foster human-robot compliance during gait training driven by an ankle-foot exoskeleton. Thus, contrarily to [10], this study controls the exoskeleton’s effects on human-robot interaction torque by comparing human-robot interaction torque-based metrics during walking trials with biofeedback and the exoskeleton’s assistance (experimental group) versus walking trials with the exoskeleton’s assistance but without biofeedback (control group). The similar results of all acquired metrics for the PRTR procedure between the experimental and control groups (Figure 4 and Figure 5) prove the balancing between the groups before the use of biofeedback regarding the outcomes of the study, fortifying the control of the exoskeleton’s effects on these outcomes.

This study determined the effects of the proposed biofeedback strategies on healthy participants as in [11]. The healthy participants were challenged to reduce their interaction with the ankle–-oot exoskeleton through active walking, aiming to improve human-robot compliance. It is hypothesized that human-robot compliance improvement will foster motor recovery in post-stroke patients. The conclusions of this study are reinforced by statistical analysis contrarily to [10]. Two-sided statistical tests were performed to conclude about the statistically significant effects of the proposed biofeedback strategies. This approach evaluated the statistically significant results in both directions (greater than or less than), preventing to exclude statistically significant results in the non-expected direction.

According to the modal training gait phase, most participants had difficulties to be compliant with the exoskeleton’s movement during plantar-flexion gait phases: between the heel-off and toe-off events, and between the heel-strike and flat-foot events, respectively. The training thresholds (Table 2) show that all healthy participants had difficulties to be compliant with the exoskeleton movement because all are in module above the baseline interaction torque of the used ankle-foot exoskeleton (baseline interaction varies between −2 Nm and 2 Nm). The non-statistically significant results regarding RMSE and delay between the reference joint angle and real joint angle (Table 3 and Table 4, *p*-value < 0.05) are expectable once the exoskeleton is assisting the user through the trajectory tracking control. These results prove that the low-level controller properly acted for the real joint angle to follow the reference joint angle, compensating possible deviations due to human-robot interaction.

Relatively to joint motion biofeedback strategy, between PRTR and TR, the experimental group increased *Performance Dir* for the training gait phase and gait cycle (Table 3, *p*-value: 0.07), and the *Performance Mag Thr* for the training gait phase (Table 3, *p*-value: 0.09). As expected, the *Performance Dir* is more affected by the joint motion biofeedback training than other metrics once this metric is directly related to the goal of this strategy that aims to encourage the users to self-control their compliance with the exoskeleton’s direction of movement. The improvements regarding *Performance Dir* and *Performance Mag Thr* suggest that the joint motion biofeedback training improved the participant–exoskeleton compliance, as mentioned by the participants, by enhancing the human-robot interaction. Between PRTR and PSTR, the experimental group increased *Performance Dir* (Table 3, *p*-value: 0.07) and *Performance Mag Thr* (Table 3, *p*-value: 0.07) for the training gait phase similarly between PRTR and PSTR. These findings suggest that motor learning related to these metrics occurred once the participants were able to maintain the increase in performance during post-training. All these results were not statistically significant; however, the related *p*-values are particularly close to the level of significance of 0.05 in comparison with the equivalent results of the control group (Table 3, *p*-value: 0.27–0.39), suggesting that the promising results reported in the experimental group were potentially caused by the biofeedback. It is hypothesized that the *p*-values will possibly reach the level of significance with longer training (more than 3 min), as commented by the participants (Table 5 and Table 6 in Q2), once the metrics did not achieve their highest values (100% for Performance, 2 Nm or below for RMS and maximum interaction torque, and −2 Nm or above for minimum interaction torque).

For the user participation biofeedback strategy, between PRTR and TR, the results of the experimental group suggest that the user participation biofeedback training significantly improved participant-exoskeleton compliance, as mentioned by the participants (Table 5 and Table 6 in Q1), by enhancing the human-robot interaction (*Performance Mag Thr* for the training gait phase significantly increased and for the gait cycle increased (Table 4, *p*-value: 0.03–5.5 × 10^−2^); minimum interaction torque (Table 4, *p*-value: 0.07); and *Performance Mag* for the training gait phase (Table 4, *p*-value: 5.57 × 10^−2^) increased). Improvements in human-robot interaction-related metrics were also verified between PRTR and PSTR (Table 4, *p*-value: 8.59 × 10^−3^–0.08), suggesting that motor learning occurred by these metrics. Therefore, 3 min training was enough to achieve motor learning with significant improvements in human-robot interaction, demonstrating the efficiency of the user participation biofeedback. On the other hand, non-significant results or large *p*-values were obtained for these metrics in the control group when compared to the experimental group (Table 4, *p*-value: 0.01–0.77). Overall, there is evidence that the promising results achieved in the experimental group were potentially caused by biofeedback.

As expected, the user participation biofeedback training affected more magnitude-related metrics, namely the *Performance Mag Thr*, *Performance Mag*, and extremes of the interaction torque than the joint motion biofeedback training. On the other hand, the joint motion biofeedback training affected more the *Performance Dir* than the user participation biofeedback training. These findings show the complementarity of both strategies, highlighting the need for sequencing both. For a low cognitive effort and effective gait training, the joint motion biofeedback training firstly may be performed and then both biofeedback strategies may be provided simultaneously to ensure that the previous motor learning remains, avoiding a decrease in *Performance Dir* between PRTR and PSTR in user participation strategy. Moreover, the participants may perform biofeedback training for the remaining gait phases (from the gait phase with the lowest performance to the gait phase with the highest performance) to achieve significant improvements regarding human-robot interaction considering all gait cycle (Table 3 and Table 4).

Contrarily to the findings of this study, the human-robot interaction torque feedback from [10] was not able to detect the motor progress of the users. On the other hand, the findings from [9,11] suggest improved motor learning upon biofeedback training, similarly to this study. However, both studies [9,11] include non-wearable BSs while the proposed biofeedback is provided through a fully wearable BS allowing ambulatory use and daily practice, which accelerates motor learning [4]. Moreover, the study [11] only addressed dorsiflexion/plantar flexion movements while sitting. Contrarily to [11], our findings show the promising impact of biofeedback for functional motor learning of gait, which is a motor task indispensable for a healthy daily living.

Furthermore, the participants demonstrated a positive appreciation concerning the auditory and vibrotactile cues and motivation to perform gait training with biofeedback, suggesting promising usability of the proposed biofeedback strategies. However, the lack of consensus concerning the timing needed to learn how to use the BS shows the need to perform a new pre-post controlled study with more participants and longer training. Regarding the usability questionnaire, most of the participants agreed with the need for physiotherapist support along with the gait training, highlighting the importance of the physiotherapist-oriented control strategies, not evaluated in [10]. This importance was reinforced by the technical person who effectively followed the participants during the experimental protocol. Studies [9,11] assessed user’s satisfaction using different questionnaires (a Likert-scale questionnaire in [11] and the Quebec User Evaluation of Satisfaction with Assistive Technology 2.0 in [9]). Similar to this study, the participants from [11] were shown to be satisfied with the experimental procedure involving biofeedback and, in [9], the acceptability of the biofeedback was rated as very high. However, the proposed wearable biofeedback solution allows the users to move freely in a space without visual reliance (contrarily to [9,11]) and the need for a treadmill (in opposition to [9]). Moreover, the proposed biofeedback does not require complex and time-consuming preparation for EMG feedback, as in [9]. Overall, this study proposes a wearable BS with promising usability and applicability for any-place gait rehabilitation.

### Limitations and Future Work

The main limitations of this work are the low number of participants and the short gait training. Furthermore, the study of the BS’s effects was limited to healthy participants and the gait phase with the lowest performance. Future work will address these limitations performing a pre-post controlled study with post-stroke patients (target end-users) and a longer gait training driven by trajectory tracking and impedance control. The quantitative effects of the physiotherapist-oriented control strategies on human-robot compliance need to be further investigated, comparing the human-robot interaction torque-based metrics between walking trials with biofeedback, the exoskeleton’s assistance, and a physiotherapist’s instructions (experimental group) versus walking trials with biofeedback and the exoskeleton’s assistance but without a physiotherapist’s instructions (control group). Moreover, further studies will include walking trials at higher walking speeds to verify the influence of walking speed on the efficacy of the biofeedback.

## 5. Conclusions

This study proposes a fully wearable BS controlled by two novel human-robot interaction-based biofeedback strategies (joint motion and user participation strategies) to boost gait rehabilitation driven by ankle-foot exoskeletons by improving patient-exoskeleton compliance and parallel control strategies to foster the physiotherapist’s involvement. The 3-min gait training with the BS and ankle-foot exoskeleton was able to improve (significant results for user participation strategy) the participants’ compliance with the ankle-foot exoskeleton movement, achieving motor learning as demonstrated in the retention trial. Controlled findings suggest that the enhanced human-robot interaction was potentially caused by the biofeedback. Moreover, the findings showed complementarity between both biofeedback strategies, as the joint motion/user participation directly/indirectly affect *Performance Dir* and indirectly/directly affect *Performance Mag*, highlighting the need for sequencing both strategies in gait training. Additionally, the participants demonstrated their acceptability and promising usability of the biofeedback strategies while walking with the ankle-foot exoskeleton. Both participants and the technical person who followed them during the experimental protocol emphasized the importance of physiotherapist-oriented control strategies. Therefore, these strategies may be powerful tools for post-stroke patients to relearn to perform a healthy gait pattern during gait rehabilitation driven by ankle-foot exoskeletons, accelerating the recovery of their ankle-foot deformities.

## Figures and Tables

**Table 1:** Gender (female/male), age (years), body mass (kg), and body height (m) for each participant. Mean and standard deviation (std) of age, body mass, and body height for the experimental and control groups.

Group	Participant	Gender (F/M)	Age (Years)	Body Mass (kg)	Body Height (m)
**Experimental**	**1**	F	27	53	1.62
	**2**	F	23	52	1.51
	**3**	M	25	76	1.82
	**4**	M	25	77	1.82
	**Mean ± Std/Mode**		25 ± 1	65 ± 12	1.69 ± 0.13
**Control**	**5**	M	25	76	1.75
	**6**	M	25	67	1.81
	**7**	M	24	76	1.70
	**8**	F	24	64	1.70
	**Mean ± Std/Mode**		24 ± 0	71 ± 6	1.74 ± 0.05

**Table 2:** Training gait phase (1, 2, 3, and 4, as illustrated in Figure 1) for the joint motion and user participation biofeedback strategies and the training threshold module of the user participation strategy (Nm) for each participant. The means and standard deviations (std) of the training threshold and modal training gait phases are shown for the experimental and control groups

Group	Participant	Training Gait Phase		Training Threshold Module (Nm)
		Joint Motion	User Participation	
**Experimental**	**1**	3	4	3
	**2**	3	1	4
	**3**	3	2	5
	**4**	2	1	4
	**Mean ± Std/Mode**	3	1	4 ± 1
**Control**	**5**	3	2	4
	**6**	4	1	6
	**7**	4	3	5
	**8**	4	2	4
	**Mean ± Std/Mode**	4	2	5 ± 1

**Table 3:** The *p*-values of the statistical tests per metric (maximum (*Max*) and minimum (*Min*) human-robot interaction torque (*Int*); RMS of *Int* for the training gait phase (TGP) and gait cycle (GC); *Performance Dir, Mag, Mag Thr* for TGC and GC; RMSE for TGP and GC; and the delay between the reference joint angle and real joint angle), considering a level of significance of 0.05 between procedures PRTR and TR and procedures PRTR and PSTR for the experimental and control groups regarding joint motion biofeedback strategy. The most representative results (*p*-value < 0.10) appear in bold

Group	Procedures	Int (Nm)		RMS Int (Nm)		Performance (%)						RMSE (deg)		Delay (ms)
		Max	Min	TGP	GC	Dir		Mag		Mag Thr		TGP	GC[M1]	
						TGP	GC	TGP	GC	TGP	GC			
**Experimental**	**PRTR vs. TR**	0.60	0.17	0.32	0.95	**0.07**	**0.07**	0.44	0.86	**0.09**	0.52	0.45	0.68	0.81
	**PRTR vs. PSTR**	0.55	0.14	0.40	0.53	**0.07**	0.27	0.53	**5.9 × 10^−2^**	**0.07**	0.11	0.45	0.48	0.80
**Control**	**PRTR vs. TR**	0.45	0.46	0.41	0.41	0.27	0.31	0.25	0.42	0.30	0.65	0.23	0.22	0.43
	**PRTR vs. PSTR**	0.45	0.14	0.90	0.19	0.26	0.71	0.81	0.34	0.39	0.68	0.69	0.53	0.40

**Table 4:** The *p*-values of the statistical tests per metric (maximum (*Max*) and minimum (*Min*) human-robot interaction torque (*Int*); the RMS of Int for training gait phase (TGP) and gait cycle (GC); *Performance Dir*, *Mag*, *Mag Thr* for TGC and GC; RMSE for TGP and GC, and the delay between the reference joint angle and real joint angle), considering a level of significance of 0.05 between PRTR and TR and between PRTR and PSTR for the experimental and control groups regarding the user participation biofeedback strategy. The most representative results (*p*-value < 0.10) appear in bold and significant results (*p*-value < 0.05) appear in italic

Group	Procedures	Int (Nm)		RMS Int (Nm)		Performance (%)						RMSE (deg)		Delay (ms)
		Max	Min	TGP	GC	Dir		Mag		Mag Thr		TGP	GC	
						TGP	GC	TGP	GC	TGP	GC			
**Experimental**	**PRTR vs. TR**	0.29	**0.07**	**0.07**	0.14	0.18	0.63	**5.57 × 10^−2^**	0.32	***0.03***	**5.5 ×10^−2^**	0.21	0.91	0.42
	**PRTR vs. PSTR**	0.26	***0.04***	**0.07**	**0.08**	**0.07**	0.39	***4.56 × 10^−4^***	0.17	***8.59 × 10^−3^***	**0.08**	0.22	0.82	0.20
**Control**	**PRTR vs. TR**	0.45	0.46	0.32	0.41	0.20	0.31	0.77	0.42	0.53	0.65	0.51	0.22	0.43
	**PRTR vs. PSTR**	0.45	0.14	***0.03***	0.19	0.25	0.71	0.08	0.34	***0.01***	0.68	0.59	0.53	0.40

**Table 5:** Comments of each participant from the experimental group regarding the performed biofeedback training with both biofeedback strategies, and comments of the technical person who followed the participants during biofeedback training regarding physiotherapist-oriented strategies.

	Comments
**Participant 1**	*The auditory cues are pleasant and they do not interfere with the external environment;* *I feel that the exoskeleton no longer pushes my foot.*
**Participant 2**	*I am more sensitive to the exoskeleton’s movement;* *I feel more synchronism between me and the exoskeleton;* *I am motivated to perform longer biofeedback training.*
**Participant 3**	*It is motivating to know my performance in real-time through the biofeedback system;* *The biofeedback helped me to understand how to walk with the exoskeleton;* *I am more active during the use of the exoskeleton;* *It is effective and ergonomic to apply the vibrotactile cue on the trained limb;* *I feel that I can achieve better results with longer biofeedback training.*
**Participant 4**	*I am motivated to use the exoskeleton with the biofeedback;* *I feel that I am more synchronous with the exoskeleton.*
**Technical person**	*It is very difficult to perceive human-robot compliance through visual inspection of the participant;* *The visual cue from the biofeedback system is an intuitive method to follow participant-robot compliance during gait training.*

**Table 6:** Answers (strongly agree (SA), agree (A), no opinion (NO), disagree (D), and strongly disagree (SD)) of each participant from the experimental group and modal answers of the group regarding usability questionnaire ((Q1) “I think that I can walk more compliantly with the exoskeleton after using the BS”; (Q2) “I need more time to learn how the BS works before I can get going with it”; (Q3) “I need to learn other things before I can get going with the BS”; (Q4) “I imagine that most people will learn to use the BS very quickly”; and (Q5) “After understanding how the BS works, I think that I need the support of a physiotherapist along with gait training”).

**Participant**	**Sentences**				
	**Q1**	**Q2**	**Q3**	**Q4**	**Q5**[M2]
**1**	SA	D	D	A	A
**2**	A	A	D	D	SA
**3**	SA	A	D	A	A
**4**	A	NO	D	D	A
**Modal answer**	SA/A	A	D	A/D	A

## Supplementary Materials

The following are available online at https://www.mdpi.com/1424-8220/20/20/5876/s1, Figure S1: Interaction torque as % of gait cycle, Table S1: Joint motion strategy-related metrics, Table S2: User participation strategy-related metrics.

## Abbreviations

This appendix comprises a glossary of the terms used in this paper, as follows:

BS	biofeedback system
GC	gait cycle
GP	gait phase
Performance Dir	user’s performance regarding the direction of human-robot interaction
Performance Mag	user’s performance regarding the magnitude of human-robot interaction
Performance Mag Thr	user’s performance regarding the magnitude of human-robot interaction with a threshold
PID	Proportional Integral Derivative
PRTR	pre-training
PSTR	post-training
RMS	Root Mean Square
RMSE	Root Mean Square Error
TGP	training gait phase
TR	training
Thr	user-adaptive threshold
