# Supplementary material for: Wearable Biofeedback Improves Human-Robot Compliance during Ankle-Foot Exoskeleton-Assisted Gait Training: A Pre-Post Controlled Study in Healthy Participants
Source: Sensors (Basel). 2020 Oct 17;20(20):5876. doi: 10.3390/s20205876 (PMC7589198; doi:10.3390/s20205876)
Supplement: Supplementary file 1 [file sensors-20-05876-s001.pdf]

sensors MDPI

Figure S1 shows the interaction torque segmented according to the reference angle's gait cycles per procedure (PRTR, TR, and PSTR) for each participant and both biofeedback strategies in the case of the experimental group.

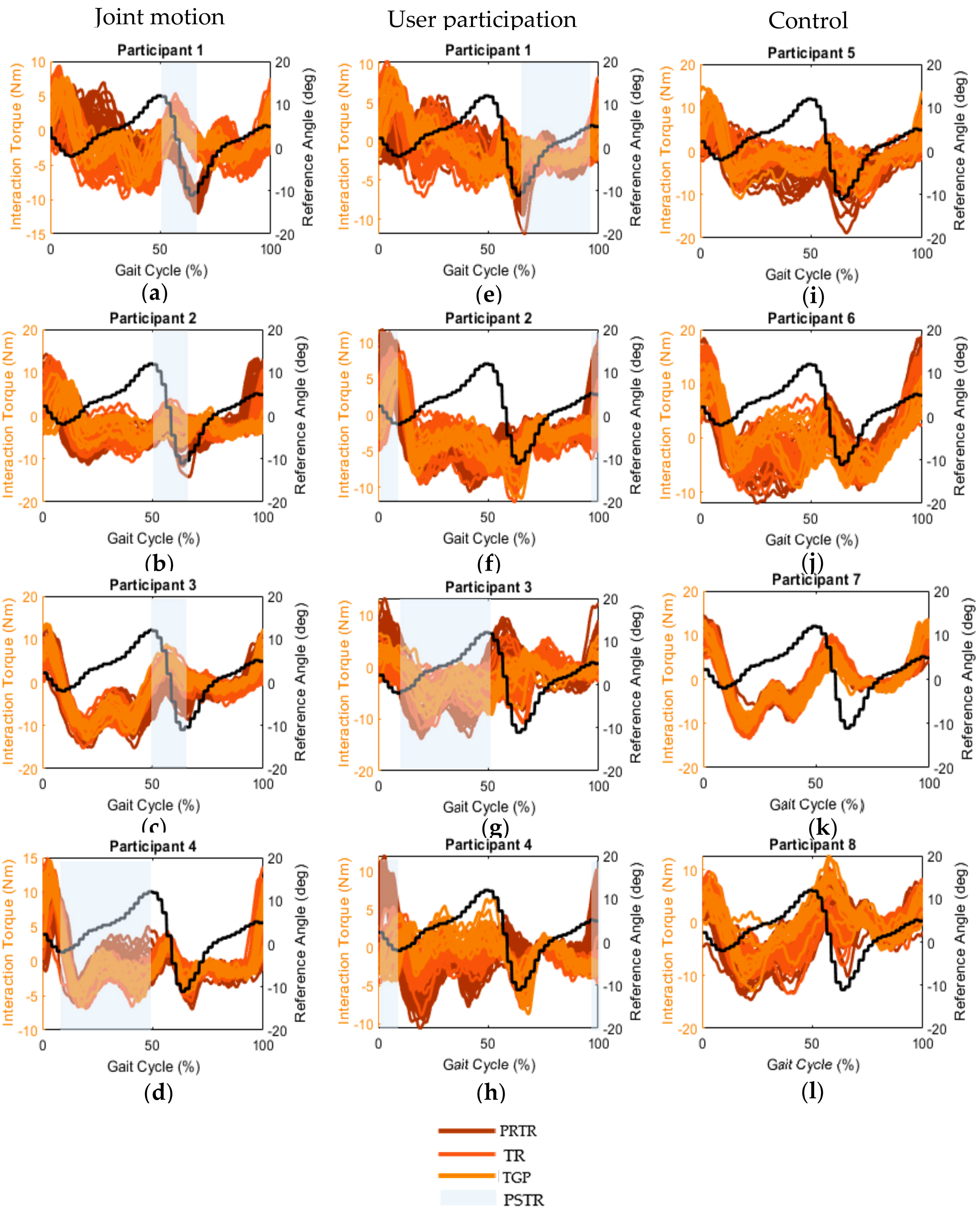

Figure S1. Interaction torque segmented according to the reference angle's gait cycles per procedure (PRTR, TR, and PSTR) of each participant of the experimental group for joint motion (**a**–**d**) and user participation biofeedback strategies (**e**–**h**), and control group (**i**–**l**).

Tables S1 and S2 present supplementary data regarding the mean and standard deviation of the acquired metrics (M1–M5) per procedure for each participant regarding joint motion and user participation biofeedback strategies, respectively. They complement the results of Figures 4 and 5, respectively.

Table S1. Mean and standard deviation per procedure (PRTR, TR, and PSTR) of the maximum (*Max*) and minimum (*Min*) human–robot interaction torque (*Int*); RMS of Int for the training gait phase (TGP) and gait cycle (GC); *Performance Dir, Mag, Mag Thr* for TGP and GC; RMSE for TGP and GC; and the delay between the reference joint angle and real joint angle for each participant regarding joint motion biofeedback strategy

Group	Participant	Procedure	Int (Nm)		RMS Int (Nm)		Performance (%)						RMSE (deg)		Delay (ms)
			Max	Min	TGP	GC	Dir		Mag		Mag Thr		TGP	GC	
							TGP	GC	TGP	GC	TGP	GC			
Experimental	1	PRTR	**4.16 ±1.17**	**-10.11 ±0.82**	**5.24 ±0.42**	**3.80 ±0.15**	**20.16 ±15.90**	**78.93 ±4.62**	20.16 ±15.90	35.81 ±8.73	20.16 ±15.90	52.62 ±6.79	3.35 ±0.12	1.72 ±0.06	241 ±4
		TR	6.38 ±0.90	-8.19 ±0.73	1.83 ±0.40	4.04 ±0.37	93.91 ±7.26	96.88 ±2.61	68.74 ±14.75	32.12 ±5.81	86.15 ±10.20	45.49 ±7.22	2.43 ±0.15	1.64 ±0.09	229 ±4
		PSTR	6.92 ±0.67	-6.56 ±1.19	1.25 ±0.40	3.27 ±0.39	92.65 ±13.98	97.63 ±2.98	89.58 ±13.92	42.57 ±9.11	92.65 ±13.98	60.97 ±8.90	2.39 ±0.10	1.55 ±0.14	232 ±7
	2	PRTR	11.09 ±1.71	-8.73 ±1.46	3.92 ±0.63	5.18 ±0.41	31.47 ±14.09	85.58 ±2.13	31.47 ±14.09	23.37 ±9.50	31.47 ±14.09	41.96 ±10.54	2.87 ±0.22	1.65 ±0.05	236 ±4
		TR	9.93 ±1.25	-7.88 ±0.81	2.18 ±0.91	4.44 ±0.51	62.41 ±28.96	91.30 ±5.91	58.19 ±29.69	28.40 ±6.56	62.41 ±28.96	55.64 ±6.73	3.21 ±0.18	1.78 ±0.11	252 ±7
		PSTR	7.45 ±1.21	-6.96 ±0.58	3.20 ±1.43	3.65 ±0.51	36.75 ±35.39	83.24 ±8.38	33.66 ±30.44	34.05 ±9.83	36.75 ±35.39	60.15 ±10.74	3.30 ±0.16	1.72 ±0.08	262 ±10
	3	PRTR	9.16 ±1.87	-11.72 ±1.52	2.78 ±1.11	5.85 ±0.63	53.11 ±30.12	89.43 ±4.83	48.06 ±25.13	31.34 ±6.81	53.11 ±30.12	51.01 ±5.30	2.85 ±0.25	1.65 ±0.10	250 ±5
		TR	7.81 ±1.11	-11.25 ±1.32	3.09 ±1.33	5.50 ±0.61	98.37 ±3.73	96.81 ±1.89	38.63 ±28.43	37.30 ±5.83	84.95 ±19.95	58.54 ±5.78	2.54 ±0.18	1.70 ±0.07	243 ±6
		PSTR	10.71 ±1.96	-11.33 ±1.17	3.41 ±1.29	5.64 ±0.29	100 ±0	96.05 ±2.44	33.00 ±25.07	39.09 ±5.67	84.53 ±22.59	56.72 ±3.98	2.51 ±0.12	1.63 ±0.05	241 ±7
	4	PRTR	8.90 ±1.00	-5.28 ±0.73	2.53 ±0.65	2.97 ±0.31	93.38 ±13.80	95.03 ±5.68	50.94 ±21.98	57.59 ±11.24	80.54 ±17.11	80.58 ±6.88	0.72 ±0.08	1.58 ±0.07	260 ±3
		TR	11.67 ±1.40	-5.44 ±0.80	2.99 ±0.69	3.73 ±0.43	99.80 ±0.58	97.25 ±1.63	37.39 ±24.21	52.37 ±10.38	80.37 ±13.04	79.67 ±5.07	0.71 ±0.08	1.56 ±0.05	256 ±4
		PSTR	12.61 ±1.21	-5.04 ±0.95	2.61 ±0.69	3.86 ±0.34	99.79 ±0.47	97.12 ±1.23	48.20 ±21.95	58.01 ±7.60	86.32 ±12.53	81.29 ±4.78	0.61 ±0.07	1.54 ±0.06	260 ±0
Control	5	PRTR	5.13 ±1.85	-9.65 ±2.66	4.47 ±2.18	4.55 ±0.73	29.94 ±28.60	86.79 ±5.43	29.40 ±27.80	33.92 ±11.69	29.94 ±28.60	54.08 ±8.76	3.08 ±0.43	1.80 ±0.21	234 ±5
		TR	7.96 ±1.93	-7.98 ±1.49	2.60 ±0.95	3.80 ±0.61	56.38 ±27.32	89.57 ±6.34	50.49 ±22.51	44.81 ±10.74	56.38 ±27.32	65.21 ±7.58	2.76 ±0.32	1.58 ±0.10	232 ±6
		PSTR	11.76 ±1.96	-8.18 ±1.65	2.75 ±1.25	4.43 ±0.49	54.11 ±33.31	90.90 ±4.36	46.84 ±29.24	40.03 ±10.82	54.11 ±33.31	61.65 ±10.16	2.61 ±0.44	1.51 ±0.12	234 ±6
	6	PRTR	13.99 ±2.33	-7.66 ±1.93	4.77 ±0.89	5.46 ±1.00	66.78 ±5.93	86.37 ±4.24	32.21 ±7.80	30.77 ±11.37	24.88 ±14.44	66.06 ±10.40	0.89 ±0.10	1.35 ±0.12	236 ±8
		TR	13.96 ±2.01	-7.60 ±1.16	4.56 ±0.86	5.36 ±0.60	78.40 ±6.42	88.27 ±6.60	31.92 ±6.64	29.78 ±9.11	23.51 ±18.10	69.48 ±8.64	0.98 ±0.17	1.36 ±0.11	234 ±5
		PSTR	11.25 ±2.10	-7.90 ±0.90	4.38 ±0.44	4.81 ±0.58	85.71 ±6.24	78.09 ±12.08	30.47 ±4.06	33.74 ±5.80	13.56 ±10.45	59.23 ±8.09	1.36 ±0.22	1.43 ±0.15	239 ±9
	7	PRTR	11.77 ±1.11	-10.00 ±0.99	2.65 ±0.74	5.36 ±0.31	81.65 ±5.20	92.91 ±1.89	78.60 ±7.54	36.54 ±3.74	81.65 ±5.20	59.67 ±4.32	1.92 ±1.64	1.47 ±0.17	223 ±40
		TR	11.12 ±1.03	-11.45 ±1.00	3.07 ±0.59	5.66 ±0.30	78.91 ±4.51	91.48 ±2.16	77.96 ±5.14	35.07 ±2.78	78.91 ±4.51	53.97 ±4.77	1.16 ±0.08	1.46 ±0.05	230 ±5
		PSTR	12.52 ±0.72	-9.51 ±1.32	4.10 ±0.63	5.47 ±0.48	77.28 ±3.61	91.48 1.80	66.78 ±7.64	34.40 ±4.44	77.28 ±3.61	59.58 ±5.16	1.30 ±0.20	1.50 ±0.08	231 ±7
	8	PRTR	6.23 ±1.35	-9.90 ±1.80	1.99 ±0.60	4.67 ±0.76	85.38 ±8.76	91.15 ±3.64	69.04 ±18.51	38.71 ±9.14	83.09 ±10.74	55.11 ±6.98	1.64 ±0.20	1.71 ±0.25	226 ±8
		TR	6.69 ±1.22	-8.63 ±1.52	1.79 ±0.41	4.39 ±0.62	85.86 ±7.70	92.35 ±3.80	78.31 ±10.96	40.90 ±6.24	84.49 ±9.02	53.51 ±7.26	1.54 ±0.23	1.57 ±0.08	230 ±7
		PSTR	8.30 ±2.99	-8.60 ±2.76	2.29 ±0.48	4.18 ±1.21	85.41 ±8.35	92.27 ±3.51	58.04 ±14.58	39.62 ±17.17	80.28 ±12.18	60.15 ±14.18	1.82 ±0.13	1.66 ±0.17	224 ±6

Table S2. Mean and standard deviation per experimental procedure (PRTR, TR, and PSTR) of the maximum (*Max*) and minimum (*Min*) human–robot interaction torque (*Int*); RMS of Int for the training gait phase (TGP) and gait cycle (GC); *Performance Dir, Mag, Mag Thr* for TGP and GC; RMSE for TGP and GC; and the delay between the reference joint angle and real joint angle for each participant regarding user participation biofeedback strategy

Group	Participant	Procedure	Int (Nm)		RMS Int (Nm)		Performance (%)						RMSE (deg)		Delay (ms)
			Max	Min	TGP	GC	Dir		Mag		Mag Thr		TGP	GC	
							TGP	GC	TGP	GC	TGP	GC			
Experimental	1	PRTR	**4.02 ±1.32**	**-6.95 ±1.56**	**3.16 ±0.43**	**2.78 ±0.31**	**100 ±0**	**78.99 ±6.53**	39.38 ±8.57	51.85 ±7.27	62.28 ±8.87	64.58 ±6.77	1.68 ±0.15	1.55 ±0.09	248 ±4
		TR	7.13 ±1.17	-4.95 ±0.78	2.04 ±0.37	2.95 ±0.39	98.37 ±2.61	88.31 ±9.00	57.41 ±17.53	48.91 ±9.94	87.83 ±14.15	65.18 ±8.00	1.65 ±0.04	1.41 ±0.05	240 ±6
		PSTR	6.99 ±1.69	-4.84 ±1.12	1.91 ±0.18	2.84 ±0.32	99.92 ±0.25	85.06 ±7.79	59.64 ±9.90	52.23 ±4.55	96.69 ±4.03	69.36 ±4.51	1.69 ±0.04	1.41 ±0.05	243 ±5
	2	PRTR	9.98 ±0.91	-8.88 ±0.78	6.83 ±0.68	5.03 ±0.25	98.81 ±2.72	85.11 ±1.74	13.40 ±4.21	21.11 ±5.31	25.32 ±3.75	43.79 ±5.55	1.05 ±0.20	1.68 ±0.07	240 ±2
		TR	7.59 ±0.97	-8.80 ±1.33	5.12 ±0.85	4.61 ±0.40	86.63 ±16.46	82.16 ±3.22	16.89 ±9.21	20.01 ±8.46	30.55 ±9.17	52.51 ±8.61	0.87 ±0.16	1.65 ±0.06	246 ±4
		PSTR	6.37 ±0.65	-8.35 ±1.39	3.97 ±0.67	4.11 ±0.34	91.95 ±10.60	83.24 ±1.90	28.90 ±13.58	22.23 ±6.17	48.70 ±14.16	59.52 ±8.04	0.74 ±0.14	1.66 ±0.09	248 ±4
	3	PRTR	7.98 ±2.14	-10.54 ±1.51	6.79 ±1.19	5.05 ±0.80	98.62 ±2.58	95.74 ±5.05	9.43 ±6.51	34.84 ±9.09	36.32 ±17.34	62.59 ±9.18	1.41 ±0.22	1.67 ±0.12	240 ±7
		TR	3.34 ±0.87	-7.97 ±2.08	4.18 ±1.51	3.31 ±0.71	99.86 ±0.76	86.63 ±6.33	28.42 ±18.00	53.30 ±8.60	70.65 ±24.29	74.19 ±9.10	1.45 ±0.21	1.72 ±0.18	254 ±6
		PSTR	4.05 ±1.30	-7.88 ±1.50	4.13 ±1.30	3.31 ±0.66	98.39 ±5.32	89.88 ±5.49	26.99 ±20.24	49.82 ±12.39	72.48 ±23.31	78.90 ±8.86	1.44 ±0.22	1.74 ±0.23	250 ±4
	4	PRTR	9.58 ±0.96	-7.09 ±1.33	6.16 ±0.81	3.59 ±0.40	92.29 ±5.62	96.30 ±2.42	21.25 ±5.45	54.55 ±6.39	33.11 ±7.45	72.68 ±4.91	1.15 ±0.17	1.36 ±0.09	232 ±5
		TR	2.96 ±1.20	-6.00 ±1.03	2.20 ±0.56	2.26 ±0.29	74.06 ±25.55	90.67 ±4.98	54.34 ±19.92	63.56 ±7.75	70.84 ±23.87	85.70 ±4.63	0.92 ±0.13	1.46 ±0.04	237 ±4
		PSTR	3.82 ±1.18	-6.07 ±1.62	2.49 ±0.59	2.32 ±0.54	51.20 ±29.96	78.14 ±14.80	40.92 ±22.99	61.68 ±15.77	50.38 ±28.72	74.32 ±16.60	0.86 ±0.15	1.40 ±0.10	240 ±3
Control	5	PRTR	5.13 ±1.85	-9.65 ±2.66	4.34 ±1.12	4.55 ±0.73	93.42 ±5.95	86.79 ±5.43	25.01 ±18.80	33.92 ±11.69	50.70 ±14.71	54.08 ±8.76	1.14 ±0.29	1.80 ±0.21	234 ±5
		TR	7.96 ±1.93	-7.98 ±1.49	2.73 ±0.54	3.80 ±0.61	92.38 ±7.63	89.57 ±6.34	50.26 ±19.58	44.81 ±10.74	80.13 ±9.72	65.21 ±7.58	0.90 ±0.27	1.58 ±0.10	232 ±6
		PSTR	11.76 ±1.96	-8.18 ±1.65	3.33 ±1.26	4.43 ±0.49	98.67 ±2.11	90.90 ±4.36	39.21 ±23.58	40.03 ±10.82	71.26 ±24.60	61.65 ±10.16	0.86 ±0.31	1.51 ±0.12	234 ±6
	6	PRTR	13.99 ±2.33	-7.66 ±1.93	9.79 ±1.91	5.46 ±1.00	98.85 ±3.83	86.37 ±4.24	9.83 ±8.85	30.77 ±11.37	24.88 ±14.44	66.06 ±10.40	1.18 ±0.16	1.35 ±0.12	236 ±8
		TR	13.96 ±2.01	-7.60 ±1.16	10.13 ±2.05	5.36 ±0.60	99.57 ±2.02	88.27 ±6.60	7.95 ±8.77	29.78 ±9.11	23.51 ±18.10	69.48 ±8.64	1.19 ±0.28	1.36 ±0.11	234 ±5
		PSTR	11.25 ±2.10	-7.90 ±0.90	9.10 ±1.75	4.81 ±0.58	100 ±0	78.09 ±12.08	1.56 ±3.09	33.74 ±5.80	13.56 ±10.45	59.23 ±8.09	1.23 ±0.35	1.43 ±0.15	239 ±9
	7	PRTR	11.77 ±1.11	-10.00 ±0.99	5.58 ±0.62	5.36 ±0.31	97.96 ±1.95	92.91 ±1.89	19.22 ±5.92	36.54 ±3.74	55.25 ±11.18	59.67 ±4.32	3.04 ±1.64	1.47 ±0.17	223 ±40
		TR	11.12 ±1.03	-11.45 ±1.00	6.56 ±0.56	5.66 ±0.30	97.43 ±2.71	91.48 ±2.16	15.09 ±4.39	35.07 ±2.78	42.59 ±7.90	53.97 ±4.77	2.22 ±0.09	1.46 ±0.05	230 ±5
		PSTR	12.52 ±0.72	-9.51 ±1.32	5.81 ±0.58	5.47 ±0.48	99.34 ±1.61	91.48 1.80	12.89 ±6.73	34.40 ±4.44	47.92 ±10.04	59.58 ±5.16	2.27 ±0.09	1.50 ±0.08	231 ±7
	8	PRTR	6.23 ±1.35	-9.90 ±1.80	3.87 ±1.67	4.67 ±0.76	96.14 ±9.00	91.15 ±3.64	30.77 ±27.57	38.71 ±9.14	58.20 ±31.93	55.11 ±6.98	1.52 ±0.28	1.71 ±0.25	226 ±8
		TR	6.69 ±1.22	-8.63 ±1.52	2.46 ±1.70	4.39 ±0.62	87.68 ±20.98	92.35 ±3.80	61.86 ±33.54	40.90 ±6.24	75.89 ±28.20	53.51 ±7.26	1.42 ±0.08	1.57 ±0.08	230 ±7
		PSTR	8.30 ±2.99	-8.60 ±2.76	5.71 ±2.56	4.18 ±1.21	98.36 ±5.43	92.27 ±3.51	17.26 ±33.00	39.62 ±17.17	30.03 ±34.02	60.15 ±14.18	1.37 ±0.41	1.66 ±0.17	224 ±6

© 2020 by the authors. Licensee MDPI, Basel, Switzerland. This article is an open access article distributed under the terms and conditions of the Creative Commons Attribution (CC BY) license (http://creativecommons.org/licenses/by/4.0/).
